# Research on Defect Detection in Lightweight Photovoltaic Cells Using YOLOv8-FSD

**DOI:** 10.3390/s25030843

**Published:** 2025-01-30

**Authors:** Chao Chen, Zhuo Chen, Hao Li, Yawen Wang, Guangzhou Lei, Lingling Wu

**Affiliations:** School of Computer Science and Engineering, Sichuan University of Science & Engineering, Zigong 643000, China; chenchao@suse.edu.cn (C.C.); 323085406210@stu.suse.edu.cn (H.L.); 323085406223@stu.suse.edu.cn (Y.W.); 323085406208@stu.suse.edu.cn (G.L.); 323085406126@stu.suse.edu.cn (L.W.)

**Keywords:** photovoltaic cell, defect detection, YOLOv8, lightweighting, FasterNet

## Abstract

Given the high computational complexity and poor real-time performance of current photovoltaic cell surface defect detection methods, this study proposes a lightweight model, YOLOv8-FSD, based on YOLOv8. By introducing the FasterNet network to replace the original backbone network, computational complexity and memory access are reduced. A thin neck structure designed based on hybrid convolution technology is adopted to reduce model parameters and computational load further. A lightweight dynamic feature upsampling operator improves the feature map quality. Additionally, the regularized Gaussian distribution distance loss function is used to enhance the detection ability for small target defects. Experimental results show that the YOLOv8-FSD lightweight algorithm improves detection accuracy while significantly reducing the number of parameters and computational requirements compared to the original algorithm. This improvement provides an efficient, accurate, and lightweight solution for PV cell defect detection.

## 1. Introduction

With the worsening of the global energy crisis and the continuous escalation of environmental degradation problems, renewable energy has gradually become an indispensable and important part of the global energy network. Among them, the application of renewable energy sources, such as solar, wind, and hydro energy, is becoming more and more widespread and has been highly emphasized by various countries [1]. Currently, the cumulative installed capacity of solar photovoltaic (PV) systems around the world shows a rapid growth trend and has become an important technological means to solve the energy shortage and reduce carbon emissions [2]. As the core component of the PV power generation system, the performance of PV cells directly determines the efficiency of power generation and the operational reliability of the system, which is the key to guaranteeing the efficient operation of PV systems [3]. However, some defects, such as cracks, hot spots and scratches, inevitably occur during the manufacturing, transportation and installation of PV cells [4]. These defects not only significantly reduce the photovoltaic conversion efficiency of PV cells and decrease the power output but also shorten their service life, leading to a significant increase in the operation and maintenance costs of PV systems [5]. Specifically, the presence of defects may require more frequent maintenance and higher repair costs, thus increasing operating costs. In addition, these defects can significantly increase the sensitivity of PV cells to environmental factors, accelerate their performance degradation and aging process, and further affect the reliability of PV systems. More seriously, certain defects may trigger cell overheating or even fire, thus posing potential safety hazards [6]. Therefore, the development of efficient and accurate defect detection methods for PV cells is of great significance to guarantee the efficient and stable operation of PV power generation systems. By quickly identifying and resolving PV cell defects, these methods can not only reduce the maintenance and operating costs of the system and extend the service life of the equipment but also significantly improve the safety and economic benefits of PV power generation. This is of great practical significance for promoting the sustainable development of renewable energy and realizing the global energy transition.

Traditional PV panel defect detection methods usually rely on manual visual inspection or basic image processing techniques. Methods based on manual visual inspection are not only inefficient but also limited by the subjective judgment of the operator, making it difficult to meet the demands of the mass production environment and real-time inspection. In traditional methods based on image processing, defective images of PV cells are analyzed mainly by manual feature selection. However, because of the complex background of photovoltaic cell images, this method is susceptible to noise interference when distinguishing defects from the background, thus significantly increasing the difficulty of removing and recognizing defect features. Tsai D.M. et al. [7] proposed using a binary clustering algorithm to perform cluster analysis on defect-free image data and employed principal component analysis to measure and evaluate each cluster. They calculated the fuzzy C-mean of the sample cluster and re-divided the worst-performing cluster into two new clusters. Defects are detected by calculating the distance from the test data to the cluster center, but this method requires manual classification each time, making it relatively complicated. Demant et al. [8] used the support vector machine method to classify and detect photovoltaic cell crack defects, training defect-free and defective samples separately. However, they extracted feature vectors from the input image utilizing local descriptors, which can lead to large errors if the descriptors are incorrect. Amaral T.G. et al. [9] proposed an unsupervised method using principal component analysis to detect photovoltaic cell fault defects by comparing the slopes of multiple photovoltaic modules. The effectiveness of these traditional machine learning methods depends heavily on feature extraction engineering, making it difficult to achieve the expected results.

For the time being, electroluminescence (EL) imaging technology is a mature, non-destructive, non-contact method for detecting defects in photovoltaic modules, and its generated image resolution is high, which can retain the characteristic information of small target defects to a great extent [10]. With the rapid development of deep learning technology, using deep learning to process photovoltaic cell EL defect images has become one of the mainstream methods. For example, the feature extraction capability of convolutional neural networks can be used to capture defect feature information in EL images and improve the detection capability for target defects. Sun et al. [11] proposed a seven-layer convolutional neural network to classify and detect photovoltaic panel defects, achieving good prediction accuracy. Demirci et al. [12] proposed a lightweight convolutional neural network architecture, which effectively reduced the redundant computation and parameter amount of the model and had strong real-time detection capability. Photovoltaic cell EL defect images have complex background interference, high image resolution, variable shapes, and diverse scales, which present great challenges for automatic defect detection. For the purpose of addressing this problem, Su et al. [13] proposed connecting the channel attention sub-network with the spatial attention sub-network to suppress background noise and highlight defect features. Additionally, some target detection models based on deep learning technology are also widely used in photovoltaic cell defect detection and identification. Compared with traditional image processing technology, deep learning target detection models can locate defects on the surfaces of photovoltaic cells more quickly and accurately, achieving efficient detection and identification.

Presently, there are mainly first-order target detection algorithms, such as SSD [14] and YOLO [15] series, and second-order algorithms, such as Faster-RCNN [16]. Although second-order algorithms have higher detection accuracy, they consume a lot of computing resources due to their complex calculations and large memory requirements. Compared with first-order algorithms, they are difficult to deploy in actual industrial production. Since first-order algorithms do not have a clear candidate region generation stage, they directly predict the bounding box and category through full image scanning or other mechanisms. Although this reduces detection accuracy, it meets the actual industrial needs, and their detection speed is greatly improved. In comparison, first-order algorithms are more suitable for deployment in photovoltaic cell industrial activities. Zhang [17] introduced a multi-scale receptive field module into the backbone network of the SSD model to enhance the receptive field of defect feature information and added a channel attention mechanism to reduce the amount of calculation while improving detection capability, but the real-time detection performance was slightly insufficient. The SSD model has certain advantages in small target detection, but its real-time performance is not as good as that of the YOLO series algorithms. Among them, YOLOv8, a significantly improved model after YOLOv5, has a good detection effect and efficiency in many application scenarios. Researchers can improve and optimize it according to their needs to improve the model.

For example, to improve the detection capability of small target defects in photovoltaic cells. Li et al. [18] added a small target detection head to the three detection heads of YOLOv5. Although this improved the recognition capability for small targets such as cracks, it also inevitably increased the model’s size and computational requirements. To minimize computation while improving accuracy, Cao et al. [19] proposed a lightweight algorithm based on the YOLOv8s model. They used a lightweight convolution called Group-shuffle Conv to replace the standard convolution of the original backbone network and introduced a bidirectional feature pyramid structure, which effectively improved the detection efficiency of photovoltaic cell defects and reduced the computational load and model size to some extent. However, considering its model size and detection speed, whether it can be applied to edge computing devices for real-time detection remains to be discussed. The large size and complexity of existing target detection models are addressed through a lightweight solution, requiring significant computing resources and hardware support for practical photovoltaic cell industrial applications, so some researchers have proposed manually designed lightweight networks. Tang et al. [20] designed and built a nine-layer convolution neural network structure, conducting experiments on numerous photovoltaic cell EL defect images generated by adversarial generative networks and traditional data enhancement to classify defect images automatically. Wang et al. [21] developed a lightweight two-stream defect detection network using frequency-doubling convolution and a two-stream parallel computing architecture to accelerate the model, resulting in a higher inference speed. However, these network structures are generally difficult to build, have a limited scope of application, and require extensive experiments to verify their feasibility.

Considering the issues in existing photovoltaic cell defect detection algorithms, this study proposes a lightweight photovoltaic cell defect detection model, YOLOv8-FSD (Fasternet Slim-neck Dysample dynamic upsampling), based on YOLOv8n. By introducing a lightweight backbone network and optimizing the feature extraction module, this model effectively reduces the model size and parameter count, while also enhancing detection speed. A series of ablation and comparative experiments confirm that YOLOv8-FSD excels in identifying various defects. Not only does it maintain high detection accuracy, but it also demonstrates superior performance in handling complex backgrounds and small target defects. This advancement strongly supports achieving high efficiency and real-time performance in photovoltaic cell defect detection, offering both theoretical insights and practical foundations for real-time monitoring applications on edge computing devices. The primary objectives of this study are as follows:

1. Replacing the backbone network involves mainly introducing a fast partial convolution to replace the original standard convolution. This aims to reduce redundant parameter calculations, computational complexity, and memory consumption.

2. Group sparse convolution and depth-separable convolution are utilized to reduce the number of parameters and computations in the model. This method enhances both the efficiency and performance of the model.

3. A dynamic upsampling operator is used to generate the sampling positions and offsets of the feature map based on the input feature map’s content and the model’s requirements. Additionally, the input feature map is divided into groups according to a specified number to enhance the diversity and representational capacity of the features.

4. A loss function constructed based on Gaussian distribution distance is used. By calculating the Gaussian distribution distance between the bounding boxes of two small target defects, the similarity between them is measured to improve the detection of small targets.

## 2. Materials and Methods

### 2.1. YOLOv8 Model

The YOLOv8 model consists of three parts: backbone, neck, and head.

Backbone: Its main function is feature extraction. It consists of the Conv, C2f, and SPPF modules. Conv is the convolution layer, which performs multi-layer convolution on the input image to extract the target feature information. C2f is mainly responsible for feature conversion and fusion. Unlike the C3 module in YOLOv5, C2f improves feature expression ability and enhances gradient flow information while reducing the complexity of model calculation by using splitting, splicing, and skip connections. SPPF is responsible for spatial pyramid pooling, which performs multi-scale pooling of input feature maps and downsampling using pooling kernels of different sizes to increase the receptive field of each feature, better understand contextual information, and reduce the loss of spatial information.

Neck: Its main function is feature fusion, further processing the features extracted by the backbone network. By adopting the Path Aggregation Network structure, it includes two parts: the Feature Pyramid Network (FPN) and the Path Aggregation Network (PAN). The FPN processes features of different scales through top-down feature fusion and bottom-up feature upsampling, while the PAN realizes multi-layer feature information fusion by introducing lateral connections and a path aggregation mechanism. The combination of the two achieves the fusion of multi-scale features and the effective integration of upper and lower feature information flows.

Head: This part is mainly responsible for converting the feature map from the neck layer into the output of target detection, namely the bounding box position and category prediction. Compared with YOLOv5, YOLOv8 adopts a decoupled head design to separate the regression and prediction subtasks, allowing the model to better handle feature maps of different scales and improve its generalization ability.

### 2.2. FasterNet

The CSPDarknet network structure employed in the original YOLOv8 algorithm is burdened with a multitude of parameters and high computational complexity, making it difficult to satisfy the real-time and lightweight demands of detecting photovoltaic cell defects in industrial settings. Reducing the computational complexity of the model, lightweight networks such as MobileNets [22] and ShuffleNets [23] often use DepthWise Convolution (DWConv) [24] to extract spatial features, achieving a lightweight network design. However, while this method reduces the amount of model computation, it also increases memory access and additional data processing operations such as pooling and shuffling. Consequently, the actual model computation speed may not increase significantly and may even decrease.

Therefore, this study selected the FasterNet [25] network to replace the backbone network in the original algorithm. This network primarily employs a simple and efficient partial convolution (PConv) [26]. By applying regular convolution to only part of the input channels, a large amount of redundant computation is reduced. This approach not only decreases computational complexity (FLOPs) but also improves computational speed (FLOPS) and reduces detection delay (latency). The calculation method is shown in Formula (1):

(1) $$\mathrm{Latency}=\frac{\mathrm{FLOPs}}{\mathrm{FLOPS}}$$

**Figure 1:**
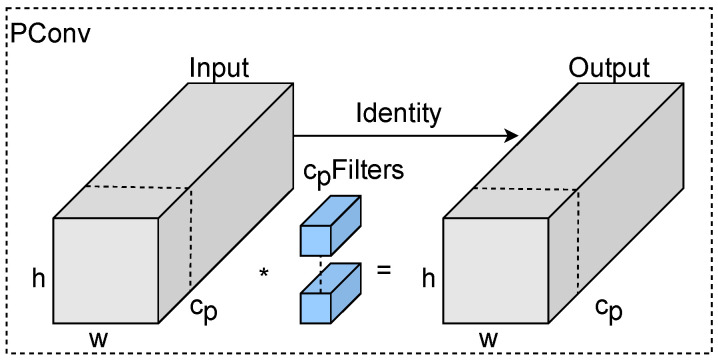
PConv structure diagram. (The “*” in the figure denotes convolution).

PConv is shown in Figure 1, and spatial feature extraction is performed only for some of the input channels (e.g., before $c_{p}$ in the figure) as a representative of the whole input channel, while the rest is kept unchanged, and the FLOPs of PConv for the number of input channels are shown in Formula (2):

(2) $$F_{PC}=h\times w\times k^{2}\times c_{p}^{2}$$

in the Equation, $F_{PC}$ is the FLOPs of the PConv, *h* is the height of the input channel, *w* is the width of the input channel, *k* is the size of the filter, and $c_{p}$ is the number of channels in the section where spatial feature extraction is performed. The FLOPs of PConv are only $\frac{1}{16}$ of the standard convolution when the number of channels of the convolution is $\frac{1}{4}$ of the original one, and PConv has less memory access, as shown in Formula (3):

(3) $$h\times w\times 2c_{p}+k^{2}\times c_{p}^{2}\approx h\times w\times 2c_{p}$$

Since PConv only performs processing for some channels, to ensure that the feature information is complete, the remaining channels should be kept unchanged and followed up with two point-by-point convolutions (PWConv), which form a convolution similar to a T-shape, which focuses more on the center position compared to the standard convolution.

**Figure 2:**
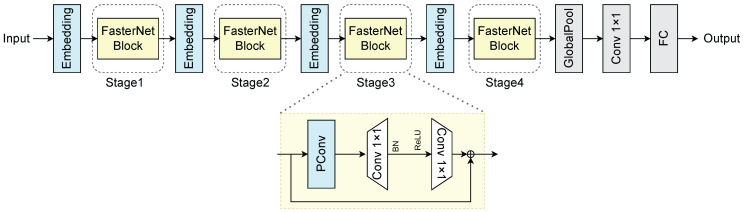
FasterNet network structure diagram.

The overall network architecture of the FasterNet module used in this study is shown in Figure 2, which is divided into four main stages, each preceded by an embedding layer (4×4 standard convolution) or a merging layer (2×2 standard convolution) to perform spatial downsampling and channel count expansion. Each stage contains a stack of FasterNet blocks, and each FasterNet block includes a PConv layer followed by two PWConv layers (or 1×1 standard convolutional). They were assembled into inverted residual blocks; the number of intermediate layer channels was extended, while shortcut connections were set up to reuse the input elements; and the choice was made to place the Batch Normalization (BN) layer and the Rectified Linear Unit (ReLU) activation layer after the intermediate layer as a way to ensure feature diversity and lower latency. Finally, a global average pooling layer, 1×1 standard convolutional, and fully connected layers were used for module feature conversion and classification, respectively.

For different application scenarios and computational resources, FasterNet has different sizes of network models. In this research, the small version of T0 is used instead of the backbone network in v8n for the actual situation of industrial activities in PV cell defect detection.

### 2.3. Slim Neck

In many lightweight network models for photovoltaic cell defect detection, Depth-wise Separable Convolution (DSConv) [27] is used throughout the network to reduce the number of parameters and FLOPs. However, its disadvantage is that part of the channel information of the input image becomes separated during the calculation process, leading to the feature extraction and fusion capabilities of DSConv being lower than those of the standard convolution.

**Figure 3:**
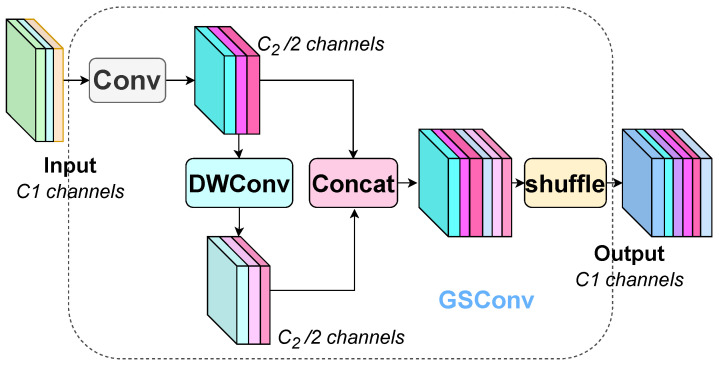
GSConv structure diagram.

Therefore, this study adopts the slim-neck [28] structure by using a new lightweight convolution called Group-Shuffle Convolution (GSConv), which combines the low computational complexity of DSConv while making its convolution output comparable to the standard convolution. The GSConv structure is shown in Figure 3. The output of the standard convolution is integrated with that of DSConv, and then the output feature information of both is concatenated. Through the channel shuffle operation, the information generated by the standard convolution is fully mixed with the information generated by DSConv.

**Figure 4:**
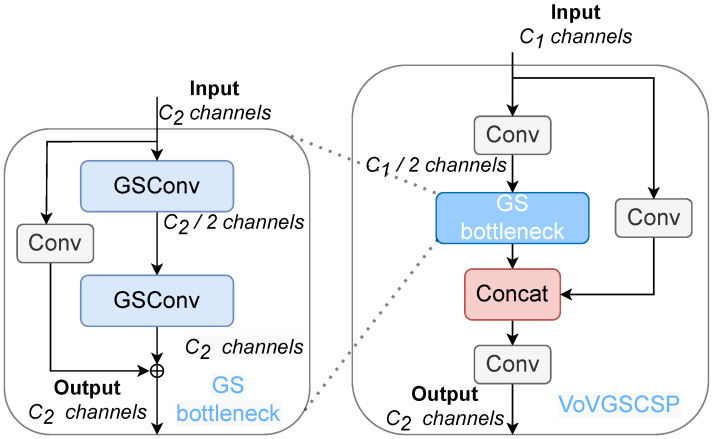
GSbottleneck modules and VoVGSCSP modules.

On this basis, the result of two GSConvs for one part of the input features and the result of one standard convolution for the other part of the input are outputted through Shortcut operation and constructed into the GSbottleneck module, as shown in Figure 4, which further strengthens the feature extraction capability. As well as by using the one-time aggregation method, the proposed cross-level partial network VoV-GSCSP structure, as shown in Figure 4, passes the output of one of the standard convolutions into the GSbottleneck module and outputs the output portion of it after stitching and combining it with the result of the other standard convolution by another standard convolution after another standard convolution operation.

When the feature map of PV cell defects is passed to the neck layer through the backbone network, its width and height are significantly reduced, and the degree of transformation is moderate. The input feature information can be transformed and reorganized using GSConv to reduce the computational burden of redundant feature information and retain more key features. Therefore, this paper adopts the slim-neck structure in the neck layer, replaces the standard convolution in the neck part of the original YOLOv8 with GSConv, and replaces C2f with the VoV-GSCSP structure, which further reduces the computational complexity of the model while maximizing the retention of the feature information of the target defects to ensure higher detection accuracy.

### 2.4. Dysample Upsampling Operator

Since the information of the feature image of photovoltaic cell defects is relatively dense, when the backbone network outputs multi-scale features, it is necessary to upsample the low-resolution features to obtain higher resolution. YOLOv8 uses the nearest neighbor interpolation method by default for upsampling. While simple and fast, this method can easily lose some semantic information from the feature images because it only considers neighboring pixel values and has a limited feature perception range. Therefore, many detection models use dynamic upsampling methods, such as Content-Aware ReAssembly of FEatures (CARAFE) [29], which employs subnets to generate dynamic convolution kernels for upsampling, albeit at the cost of increased inference time and computational expense. Compared to other dynamic upsampling methods, the Dysample upsampling operator [30] selected in this study has smaller parameters, lower inference delay, and reduced memory usage. While ensuring the effectiveness of dynamic upsampling, it also maintains lower overhead.

**Figure 5:**
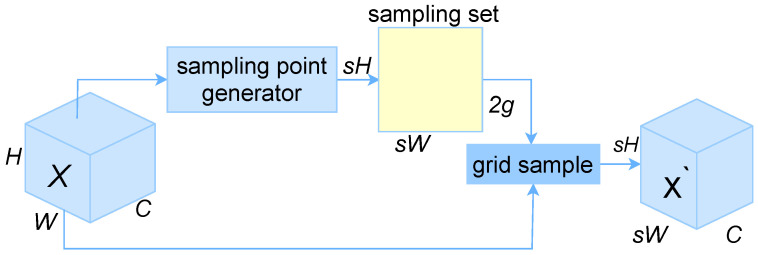
Dysample dynamic upsampling.

The process of dynamic up-sampling in Dysample, as shown in Figure 5, is that for a given input feature map of size $C\times H_{1}\times W_{1}$ (where *C* is the number of input channels, *H* is the channel height, and *W* is the channel width) and a sampling set *S* of size $\;2\times H_{2}\times W_{2}$ (the first “2” represents the location coordinates), the grid_sample function resamples the assumed bilinearly interpolated *X* using the positions in the *S* to generate the upsampled feature $X^{\prime }$ of size $\;C\times H_{2}\times W_{2}$, which is defined as shown in Equation (4):

(4) $$X^{\prime }=\mathrm{grid\_sample}\left(X,S\right)$$

in the equation, $X^{\prime }$ is the up-sampled feature, *X* is the input feature map after bilinear interpolation, *S* is the sampling set, and grid_sample is the sampling function.

**Figure 6:**
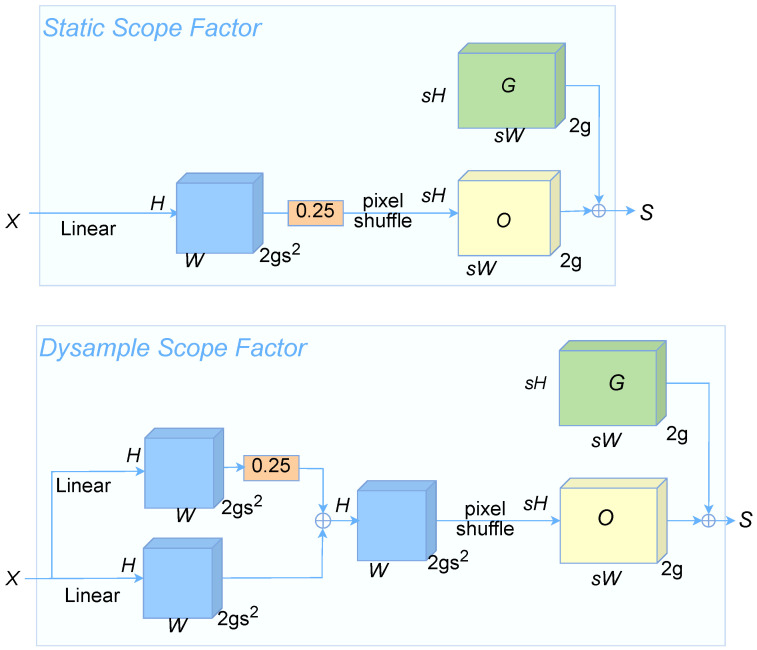
Dysample sample generator.

The sampling set *S* is generated by the sampling generator, as shown in Figure 6, being divided into static and dynamic range factors. The upper box in the figure is the static range factor for the feature map *X* whose input feature size is $C\times H_{1}\times W_{1}$. First, use a linear layer with input and output channel numbers *C* and $2s^{2}$, respectively, to generate an offset *O* of size $\;2s^{2}\times \;H\;\times \;W$. Then, the output is 2×sH×sW through the shuffle operation, and finally the final sampling set *S* is formed by the sum of the generated offset *O* and the original sampling set *G*. The process definition is as shown in Equation (5):

(5) $$\begin{array}{cc} \; & O=\mathrm{linear}(X)\\ \; & S=G+O \end{array}$$

The static range factor is designed to capture stable, global patterns in input features and is suitable for tasks where features do not change with context or input. In contrast, the dynamic range factor, shown in the lower box of the figure, achieves dynamic feature extraction through a more complex path. The input feature *X* is linearly transformed to generate intermediate features. Two parallel branches are then used to process these features, enhancing their flexibility and adaptability. Each branch undergoes weight scaling and shuffle operations, ultimately generating dynamic features *G* and *O*. This design allows the dynamic range factor to capture changes in input features across space or context, making it particularly suitable for tasks requiring the extraction of complex local information.

The Dysample dynamic upsampling operator used in this study replaces the upsampling method in the original YOLOv8n to achieve effective sampling of photovoltaic cell defect feature maps. This replacement expands the feature perception range, improves the quality of feature maps, and enhances the ability to detect target defects. Additionally, the Dysample operator maintains good computational complexity and model performance.

### 2.5. Wasserstein Distance

There are many small target defects in photovoltaic cells, such as cracks and stains. The Intersection over Union (IoU) index between the target prediction box and the real box is very sensitive to the position deviation of the small target. When the deviation between the prediction box and the real box is large, the IOU value drops significantly. This makes the traditional Complete Intersection over Union (CIoU) loss function used in YOLOv8n, which only considers the overlapping part, less effective in distinguishing the similarity between different small targets, thereby reducing the performance of small target defect detection in anchor-based detectors. Therefore, a loss function based on the Normalized Wasserstein Distance (NWD) design is used to improve the detection of similar small target defects [31]. NWD mainly calculates the similarity between two small targets by the corresponding Gaussian distribution and measures the similarity of their distributions even without overlapping parts.

By using the Wasserstein distance of optimal transport theory to calculate the distribution distance between small target defects, the Gaussian two-dimensional distributions $N_{a}$ and $N_{b}$ are modeled for the bounding box $A=(cx_{a},cy_{a},w_{a},h_{a})$ and the bounding box $B=(cx_{b},cy_{b},w_{b},h_{b})$; the second-order Wasserstein distance can be defined as shown in Equation (6):

(6) $$W_{2}^{2}\left(N_{a},N_{b}\right)={∥\left({\left[cx_{a},cy_{a},\frac{w_{a}}{2},\frac{h_{a}}{2}\right]}^{T},{\left[cx_{b},cy_{b},\frac{w_{b}}{2},\frac{h_{b}}{2}\right]}^{T}\right)∥}_{2}^{2}$$

in the equations, the parameters (cx,cy), *w*, and *h* refer to the center coordinates, height, and width of the corresponding bounding box, respectively. The parameters $N_{a}$ and $N_{b}$ represent the respective Gaussian two-dimensional distributions.

The normalized NWD is obtained by normalizing the $W_{2}^{2}$ exponential form, and the loss function designed based on it is as shown in Equation (7):

(7) $$L_{NWD}=1-\exp\left(-\frac{\sqrt{W_{2}^{2}\left(N_{a},N_{b}\right)}}{C}\right)$$

in the equation, $L_{NWD}$ is denoted as the loss function based on the NWD design, and *C* is a constant that is closely related to the dataset.

This study uses a loss function designed with NWD as an indicator to replace the CIoU loss function in YOLOv8n, thereby reducing the sensitivity of the IoU to the position deviation of dense small target defects in photovoltaic cells and improving the detection ability of the model for small target defects.

### 2.6. Dataset

**Figure 7:**
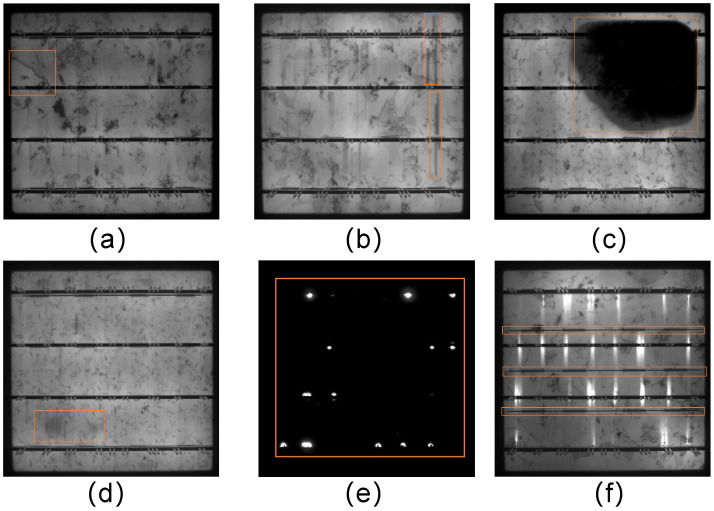
Photovoltaic cell defect category images: (**a**) crack (crack), (**b**) finger interruption (finger), (**c**) black core (black_core), (**d**) thick line (thick_line), (**e**) short tail defect (short_circuit), (**f**) and horizontal dislocation (horizontal_dislocation). (The orange sections in the figure mark the current locations of defects).

The PVELAD dataset [32] used in this study is a photovoltaic cell defect dataset jointly released by Hebei University of Technology and Beihang University. The photovoltaic dataset used in this study removed the defects with fewer instances in PVELAD and retained six major defects, as shown in Figure 7, including crack, finger, black core, thick line, short circuit, and horizontal dislocation, with a total of 4500 images. The dataset was divided into the training set, validation set, and test set at a ratio of 7:2:1. The training set contained 3150 images, the validation set contained 900 images, and the test set contained 450 images. The defects in the images were annotated using Labeling software. The labeling software is available from “https://github.com/HumanSignal/labelImg” (accessed on 15 November 2024).

## 3. Results

### 3.1. YOLOv8-FSD Lightweighting Model

**Figure 8:**
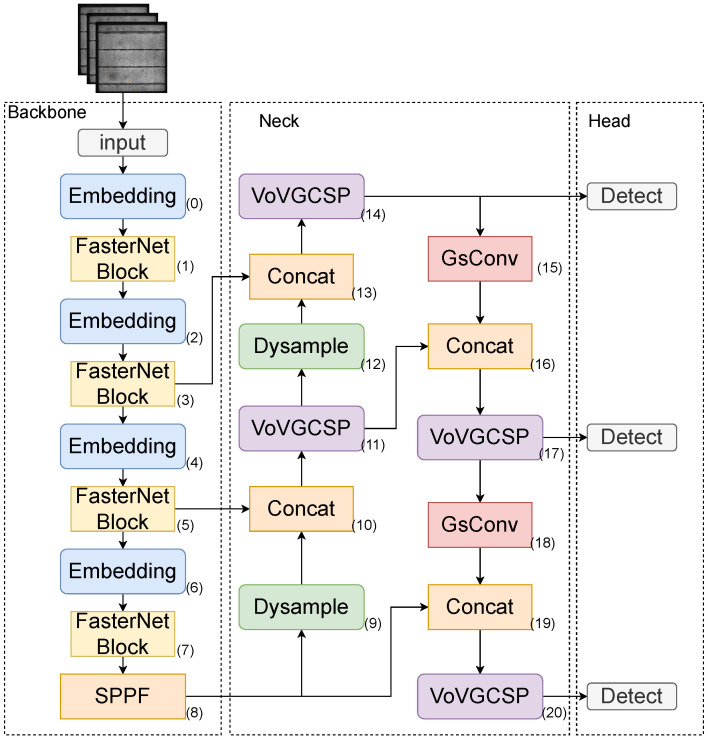
YOLOv8-FSD structure diagram.

The YOLO algorithm models for photovoltaic cell defect detection exhibit high detection accuracy and effectiveness. However, these models are large in size, and their inference detection speed is slow under limited computing resources, making it challenging to apply them in actual photovoltaic cell industrial activities. This study addresses this issue by proposing a lightweight model based on improvements to YOLOv8n, named YOLOv8-FSD. The structure diagram of the model is shown in Figure 8.

The number of parameters and computational complexity of the model are reduced by replacing the original YOLOv8n backbone network with FasterNet. An embedding layer is placed before each FasterNet module to convert the output data of the previous layer into high-dimensional features suitable for processing in subsequent layers. The partial convolution adopted by each FasterNet module reduces redundant calculations while retaining feature diversity. After four stages of convolution processing, the spatial pyramid pooling layer is used to enhance feature extraction using multi-scale feature maps. The data are then passed to the neck module. In the neck layer, a dynamic upsampling operator with reduced computational overhead is used. The sampled output features are connected with the output features of the fifth FasterNet layer to serve as input for the next layer, effectively preserving important features. This strategy is similarly applied to other connection layers in the neck. The next layer uses the VoVGSCSP module (11th layer) to reduce computational load while maintaining gradient flow, thereby improving training efficiency and model performance. Dynamic upsampling is performed again at layer 12, connecting its output with the output of the FasterNet module in layer 3. The data then pass through the VoVGSCSP layer (layer 14) to reduce computational load, and the result is input into the detection layer. Simultaneously, the result of layer 14 passes through the GSConv layer (layer 15) for feature extraction. Its output is connected with the result of layer 11 and input into the VoVGSCSP layer (layer 17). The result is input into the detection layer, followed by another GSConv operation at layer 18. Its output is connected to the SPPF layer at layer 8 for a VoVGSCSP operation (layer 20), and finally, the result is input into the detection layer. Detection layers are present at different stages in the head layer, ensuring the model can effectively detect multi-scale targets and improve detection accuracy.

### 3.2. Experimental Configuration

The hardware experimental environment used in this study is as follows: The CPU uses Intel(R)Xeon(R) Gold 5218 CPU @ 2.30 GHz (Intel, Santa Clara, CA, USA), the graphics card uses NVIDIA Quadro RTX 5000 (Nvidia, Santa Clara, CA, USA), and the video memory size is 16 GB.

The software experimental environment is as follows: Windows 10, 64-bit operating system, Pytorch version 2.12, CUDA version 12.1, and Python environment 3.9.

The model parameters are set as follows: the input image size is 640 × 640, the number of training rounds is 200, the initial learning rate is set to 0.01, cosine learning rate decay is used, the momentum is set to 0.937, the batch_size size is set to 32, and the Auto optimizer is used for parameter tuning.

### 3.3. Model Performance Evaluation Metrics

The common evaluation indicators used in the YOLO series are Precision (P), Recall (R), Average Precision (AP), mean Average Precision (mAP), and other indicators. The calculation method is as follows [33,34]:

(8) $$\begin{array}{c} P=\frac{TP}{TP+FP} \end{array}$$

(9) $$\begin{array}{c} R=\frac{TP}{TP+FN} \end{array}$$

(10) $$\begin{array}{c} \mathrm{AP}=\int_{0}^{1}P\left(R\right)\;dR \end{array}$$

(11) $$\begin{array}{c} \mathrm{mAP}=\frac{\sum_{j=0}^{n}AP(j)}{n} \end{array}$$

Among them, TP represents a true example, FP represents a false positive example, FN represents a false counterexample, *n* represents the category, and the defect category in this experiment is 6.

In order to validate the performance of the YOLOv8-FSD algorithm model, this experiment uses the average detection accuracy (mAP@0.5) with a step size of 0.5 in percentage and frames per second (FPS) to measure the performance of the model in detecting defects, and it uses the number of floating-point operations per second (GFLOPS) and parameter sizes (parameter) in millions (Ms), while the size of the model weights file (weights), which is in MB, is used to measure the size of the model.

### 3.4. Ablation Experiment

In this study, a lightweight algorithmic model, YOLOv8-FSD, is proposed to address the computational resource constraints and real-time requirements in the industrial inspection of photovoltaic cells. The model introduces the FasterNet backbone network to reduce computational complexity and increase speed, adopts the slim-neck structure to further decrease the number of parameters and computations, and utilizes Dysample upsampling to expand the feature perception range, thereby improving the quality of feature maps. Additionally, by incorporating the Wasserstein distance loss function to enhance defect detection on small targets, the model ensures both high efficiency and accuracy.

The effectiveness of the model improvements was evaluated through relevant ablation experiments conducted in this study. As shown in Table 1, in Experiment 1, the original backbone network was replaced with FasterNet, resulting in a 1.5% decrease in detection accuracy mAP. However, the GFLOPs and Params decreased by 37% and 42%, respectively, and the model volume (Weight) was reduced by 38.3%. This trade-off of a slight loss in detection accuracy for a significant reduction in computation, parameters, and volume also led to a modest increase in the model’s detection speed. Experiment 2 introduced the slim-neck module, which improved accuracy by 1.7%, while also reducing the computational amount, parameter count, and volume. However, this resulted in a decrease in detection speed. In order to further improve the detection accuracy, the “mis-sampling” module and the NWD-based loss function were introduced in Experiments 3 and 4, respectively. Both improvements yielded increases in detection accuracy of 1.9% and 2%, respectively, without significantly affecting computation, parameter count, or model volume, indicating that these modules did not add extra computational costs. In Experiment 5, the slim-neck structure was added to Experiment 1, leading to a modest 0.3% increase in accuracy while further reducing the computational volume and parameter count by 17.6% and 12.6%, respectively, compared to Experiment 1. Experiment 6 then added the Dysample module to Experiment 5, improving detection accuracy by 1.5% without significant changes in computation, parameter count, or volume. Finally, the YOLOv8-FSD model, obtained by adding the NWD-based loss function in Experiment 6, increased the model’s ability to detect small target defects, improving accuracy by 1.9% while maintaining similar computational and parameter levels. The final lightweight model, YOLOv8-FSD, for PV cell defect detection, demonstrated a 2.2% improvement in detection accuracy compared to YOLOv8n, along with reductions in computation and parameter count by 48.2% and 48.7%, respectively, and a volume reduction of 38.3%. Additionally, the detection speed reached 104.5 FPS. Thus, the improved fusion of these methods not only meets the lightweight requirements for PV cell defect detection but also significantly enhances detection accuracy. This algorithmic model offers a significant advantage in actual PV cell industrial activities, particularly under conditions of limited computational resources.

## 4. Discussion

### 4.1. Comparison Experiment

The effectiveness of the YOLOv8-FSD algorithm for photovoltaic cell defect detection was further verified through comparative experiments. The proposed model was compared with the basic YOLOv8n model and other target detection algorithms, including YOLOv7-tiny, YOLOv8n+MobileViT, YOLOv8n+ShuffleNet-V2, and YOLOv8s. These models were trained and tested using the same photovoltaic cell defect dataset and model parameters and were evaluated using indicators such as mean average detection accuracy (mAP), floating-point operations per second (GFLOPs), parameter quantity (Params), model weight file size (Weight), and frames per second (FPS). The results are presented in Table 2.

The model in this study demonstrates a detection accuracy (mAP) of only 0.6% lower than those of YOLOv5s and YOLOv8s, which have the highest detection accuracy. However, its floating-point operations per second (GFLOPs), parameter count (Params), and weight file size (Weight) are much lower than those of the basic v5s and v8s models. When comparing the experimental results of replacing the YOLOv8n backbone network with the mainstream lightweight network modules MobileViT, ShuffleNetV2, and FasterNet, it is observed that the former two indeed reduce the amount of computation and computational complexity of the model, achieving a lightweight effect. However, their detection accuracy (mAP) decrease by 2.5% and 4.5%, respectively, whereas the FasterNet network only decreased by 1.5%. The MobileViT and ShuffleNetV2 network models use deep convolution to extract spatial features, resulting in increased memory access while reducing the model’s computational workload, thereby decreasing the model’s detection speed. In contrast, the partial convolution used by the FasterNet network does not incur this extra overhead, leading to an increased detection speed by 3.3 FPS, which further validates the effectiveness of choosing FasterNet as the backbone network. Comparison with the detection results of the two small-size and tiny models of YOLOX reveals that the detection accuracy (mAP) values are 5.5% and 4.3% higher, respectively. Additionally, the number of floating-point operations and parameters of the model are smaller than those of the two YOLOX models. Furthermore, the detection speed values are also 28.86 FPS and 12.85 FPS higher, respectively.

Overall, the method proposed in this paper achieves a high detection accuracy of 92.9% for PV cell defects, meeting the needs of practical industrial applications. In terms of evaluation metrics, including model parameters, computational complexity, weight file size, and inference speed, the method demonstrates significant advantages over existing models for applications in resource-constrained environments. Furthermore, it exhibits the efficiency and lightweight potential necessary for deployment on edge computing devices.

### 4.2. Detection Effect

**Figure 9:**
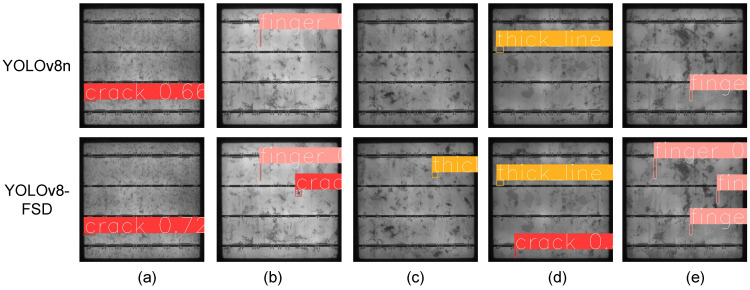
Comparison of model detection results before and after improvement. (Figures (**a**–**e**) show the final detection results of the two models, in which the red detection box is for crack defects, the pink detection box is for finger defects, and the orange detection box is for thick line defects).

In an effort to validate the effectiveness of the proposed detection model, the same set of defect images was used as a test dataset for both our YOLOv8-FSD model and the original YOLOv8n model. A comparative analysis of the detection results is presented in Figure 9. It is evident that YOLOv8n exhibits a higher rate of missed detections compared to YOLOv8-FSD. For instance, in both (b) and (d), one crack defect was undetected; in (c), a thick line defect was overlooked; and in (e), two finger-like defects remained undiscovered. Moreover, even when YOLOv8n successfully localized a defect, its confidence in classifying the defect type was lower than that of the improved model. Taking (a) as an example, YOLOv8n achieved a confidence level of merely 0.66 (indicating a 66% likelihood of the defect being a crack), while the improved model reached 0.72 for the same defect. These observations suggest that the improved model possesses stronger feature extraction capabilities, enabling it to capture more comprehensive defect information, particularly for small target defects.

## 5. Conclusions

Considering the constraints posed by computing resources, real-time performance, and detection speed in the current photovoltaic cell industry when utilizing deep learning technology for detecting defects on photovoltaic cell surfaces, this study introduces a lightweight algorithm model YOLOv8-FSD based on YOLOv8n. Through a series of enhancements to the model structure, significant reductions in volume and computational complexity are achieved alongside further improvements in detection accuracy. This is primarily achieved by replacing the backbone network in the original YOLOv8n with a FasterNet network featuring fewer parameters and computational complexities. The core innovation lies in utilizing an efficient partial convolution PConv, which diminishes redundant calculations by convolving only a portion of the input channel, thus reducing computational complexity without demanding extensive memory access. This enhancement is substantiated by the ablation experiment results in Table 1. Additionally, the slim-neck structure is incorporated into the original network model’s neck structure, and GSConv convolution is employed to further curtail computational load while preserving target defect characteristics to a significant extent. Simultaneously, a lightweight dynamic upsampling Dysample replaces the original upsampling method in the neck structure, broadening the feature perception range, enhancing feature map quality, and augmenting the model’s ability to detect photovoltaic cell defects. Given the prevalence of dense small target defects in photovoltaic cell defect images, a loss function based on Gaussian distribution distance is ultimately adopted to bolster small target defect detection capabilities. Through various experiments, it is demonstrated that the YOLOv8-FSD algorithm model in this study achieves an average detection accuracy improvement of 2.2% compared to the original algorithm model, with reductions in computation and parameters of 48.2% and 48.7%, respectively. Moreover, the model’s volume is reduced by 38.3%. Additionally, the detection speed of the model reaches 104.5 FPS, which meets the practical application requirements for photovoltaic cell defect detection. Consequently, even under resource-constrained conditions, the algorithm model in this study maintains high detection accuracy and performance in photovoltaic cell defect detection, exhibiting promising feasibility and practicality for actual deployment in photovoltaic cell defect detection tasks.

Regarding the practical application of the model in this paper, it can run on low-cost GPUs or other devices to ensure a fast response, even with limited computational resources. These devices need edge computing capabilities to adapt to on-site detection needs, such as being mounted on a drone. In actual deployment, the model should be tested and tuned in the field to ensure its robustness and accuracy under different conditions. A continuous monitoring and feedback mechanism should be established to optimize the model based on actual needs.

The next step of this research will focus on expanding the diversity and size of the dataset to cover more defect types and environmental conditions, improving the model’s generalizability. Additionally, the model will be deployed on edge computing devices to facilitate the real-time inspection of photovoltaic cells in the industry, with continuous optimization based on feedback results.

## Figures and Tables

**Table 1:** Ablation experiment results.

Model	FasterNet	Slim_Neck	Dysample	NWD_Loss	mAP@0.5/%	GFLOPs	Params (M)	Weight (MB)	FPS
v8n	✗	✗	✗	✗	90.7	8.1	3.0	6	163
Experiment-1	✓	✗	✗	✗	89.2	5.1	1.75	3.7	166.3
Experiment-2	✗	✓	✗	✗	92.4	7.3	2.8	5.6	125.4
Experiment-3	✗	✗	✓	✗	92.6	8.1	3.02	6	126.7
Experiment-4	✗	✗	✗	✓	92.7	8.1	3.01	6	149
Experiment-5	✓	✓	✗	✗	89.5	4.2	1.53	3.2	127.2
Experiment-6	✓	✓	✓	✗	91	4.2	1.54	3.2	105.4
YOLOv8_FSD	✓	✓	✓	✓	92.9	4.2	1.54	3.2	104.5

**Note:** “✓” indicates that the module is added, while “✗” indicates that it is not.

**Table 2:** Comparison experiment results.

Model	mAP@0.5/%	GFLOPs	Params (M)	Weights (MB)	FPS
YOLOv5-s	93.5	15.8	7	14.5	136.9
YOLOv8-n	90.7	8.1	3.0	6	163
YOLOv8-s	93.5	28.4	11.13	21.5	133.7
YOLOv7-tiny	77.0	13.1	6	12.3	162.3
YOLOv8n+MobileViT	88.2	5.3	1.18	2.7	65.5
YOLOv8n+ShuffleNetV2	86.2	5	1.71	3.7	137.2
YOLOv8n+FasterNet	89.2	5.1	1.75	3.7	166.3
YOLOX_s	87.4	26.77	8.94	-	75.64
YOLOX_tiny	88.6	15.25	5.03	-	91.65
YOLOv8_FSD	92.9	4.2	1.54	3.2	104.5

## Data Availability

Data are contained within the article.

## Abbreviations

The following abbreviations are used in this manuscript:

**Abbreviation**	**Explanation**
YOLOv8-FSD	YOLOv8 Fasternet Slim-neck Dysample dynamic upsampling
mAP@0.5%	Average detection accuracy with a step size of 0.5
GFLOPs	Number of floating point operations per second
Params	Number of parameters
Weight	Model weight file size
Latency	Detection delay
FLOPs	Computational complexit
FLOPS	Computational speed
$F_{PC}$	FLOPs of the PConv
*h*	Height of the input channel
*w*	Width of the input channel
*k*	Size of the filter
$c_{p}$	The number of channels for spatial feature extraction
$X^{\prime }$	Up-sampled feature
*X*	Input feature map after bilinear interpolation
*S*	Sampling set
*O*	The resulting offset
*G*	Original sampling set
$N_{a}$,$N_{b}$	Gaussian distribution of the bounding boxes a and b
$cx_{a},cy_{a},w_{a},h_{a}$	Center coordinates, height, and width of the bounding box a
$cx_{b},cy_{b},w_{b},h_{b}$	Center coordinates, height, and width of the bounding box b
$L_{NWD}$	The loss function based on the NWD design
*C*	A constant that is closely related to the dataset
*P*	Precision
*R*	Recall
AP	Average Precision
mAP	mean Average Precision
TP	True example
FP	False positive example
FN	False counterexample
*n*	Number of categories
